# Seasonal antioxidant and biochemical properties of the Northern Adriatic *Pecten jacobaeus*

**DOI:** 10.1371/journal.pone.0230539

**Published:** 2020-03-18

**Authors:** Natalija Topić Popović, Blanka Beer Ljubić, Ivančica Strunjak-Perović, Sanja Babić, Vanesa Lorencin, Margita Jadan, Lara Čižmek, Daniel Matulić, Krunoslav Bojanić, Rozelindra Čož-Rakovac

**Affiliations:** 1 Laboratory for Aquaculture Biotechnology, Ruđer Bošković Institute, Zagreb, Croatia; 2 Centre of Excellence for Marine Bioprospecting-BioProCro, Ruđer Bošković Institute, Zagreb, Croatia; 3 Faculty of Veterinary Medicine, University of Zagreb, Zagreb, Croatia; 4 FLAG Istarski Švoj, Pazin, Croatia; 5 Faculty of Agriculture, University of Zagreb, Zagreb, Croatia; National Institute of Child Health and Human Development (NICHD), NIH, UNITED STATES

## Abstract

The present work is the first study of Mediterranean scallop (*Pecten jacobaeus*) biochemical properties, antioxidant defenses, and free radical scavengers during the yearly seasons in the Northern Adriatic, off Istria. Scallop nutrient reserves (glucose, triglyceride, and cholesterol) in four tissues under examination were positively correlated and were predominant in digestive gland and gonad. The muscle energy maxima were in correlation with the maximum fall gonosomatic index (GSI), when diatoms and coccolithophorids thrive. The decrease of GSI in summer might be related to the spawning or resorption of gametes. Summer also revealed elevated levels of glucose in gonad and digestive gland, while muscle glucose and cholesterol significantly varied in spring *vs*. winter samples. In relation to the diatom seasonal abundance, carotenoids, namely astaxanthin peaks were found in digestive gland, which, being stimulators of calcium transport over cell membranes, could have contributed to the high digestive gland levels of calcium in winter. In winter, total antioxidative status (TAS) of scallop tissues was 3-fold higher than in other seasons, particularly in digestive gland, having a significant correlation with magnesium, a regulatory tool in oxidative processes. The winter maxima of TAS and thiobarbituric acid reactive substances TBARS in relation to summer maxima of glutathione peroxidase and superoxide dismutase in digestive glands indicate to a decrease in antioxidant defense during cold months, and are related to the accumulation of lipid peroxidation products (such as malondialdehyde) in digestive gland of scallops. Although the increased susceptibility to oxidative stress could be attributed to winter temperature, other factors such as the gonad maturation, availability of food supply, and salinity might counteract that effect. The seawater alterations of salinity, temperature and water quality are in relation to the river Po influx, which is very likely to influence the physiological and biochemical responses of scallops in the Northern Adriatic.

## Introduction

Mediterranean scallops (*Pecten jacobaeus* L.) are endemic to the Mediterranean, with the Spanish shores as their westernmost limit [1]. They naturally occur in exploitable quantities only in the Northern Adriatic Sea, where they tend to be overfished, particularly off the north-west coast of Istria, Croatia [2]. Benthic dredging particularly contributed to scallop decline [3]. The dredge was introduced in the Adriatic Sea fishing in the 1960s and it dramatically increased their exploitation [4].

Mediterranean scallops have a flat upper brownish shell and a convex whitish lower shell with 15–18 accentuated ribs, and a maximum shell length of 15 cm. They are hermaphrodites achieving sexual maturity at 5–6 cm of shell length [5,6]. As the sexes differentiate and gametogenesis proceeds, gonads attain typical cream or orange color, respectively. The gonad of sexually mature scallops is directly attached to the anterior margin of the adductor muscle. Ventral to the gonad are the two large gills, attached to the adductor muscle via suspensory membranes. The stomach is situated within the digestive gland [7]. The striated adductor muscle, however, is the main reason for a commercial exploitation of Mediterranean scallops, and aquaculture efforts in the Adriatic Sea are directed towards potential farming of scallops for gastronomic purposes.

In the Southern Adriatic (Mljet), *P*. *jacobaeus* attain the length of 100 mm after their fourth year of life, whereas in the mid-Adriatic (Krka) shells reach the length of 100 mm only after their fifth year [8,9]. However, in the Italian part of the Northern Adriatic they attain that length in two years [4], while on the Croatian side they need three years for 100 mm [8]. Therefore, as variation of growth of *P*. *jacobaeus* is location dependent, their biochemical and antioxidative properties could also be related to the particular location.

Although their growth and age composition in the Adriatic Sea are well investigated [8,9], as well as the effects of exposure to aquatic contaminants [10,11], and tissue contents of some macrominerals [12], the antioxidative capacity and lipid peroxidation of the North Adriatic scallops have not yet been evaluated. Albeit used mostly for assessing the effects of xenobiotics, biological responses or biomarkers can, due to their rapid response to stress [13], also be regarded as the indicators for long-term ecological effects on bivalves [14]. In the region of the North Adriatic Sea, the seawater alterations of salinity, temperature, and water quality are related to the river Po influx, which might influence the physiological and biochemical responses of bivalves. The capacity of scallops to adapt to possible seawater variations is of utmost significance as they might negatively impact their physiology, growth, survival and reactions to stress [15,16]. To that end, and for the lack of information of the effects of seasonal changes on scallop responses, four tissues of Istrian Mediterranean scallops of both sexes (gills, digestive gland, gonads, muscle) have been studied over yearly seasons for selected biochemical properties, antioxidant defenses and free radical scavengers. These included tissue magnesium (Mg), calcium (Ca), glucose (GLU), triglyceride (TRIG), and cholesterol (CHOL) concentrations, activities of lactate dehydrogenase (LDH), superoxide dismutase (SOD), glutathione peroxidase (GPx), total antioxidant status (TAS), malondialdehyde (MDA) and total carotenoids and astaxanthin contents, with the following rationale:

Tissue Mg and Ca, aside from being formative shell elements, play a significant role in maintaining ionic homeostasis of clams along the salinity gradient [17]. They also impact the prostaglandin release from tissues and are thus related to reproduction control in bivalves [18,19]. Tissue protein concentrations are crucial for the protein catabolism as an alternative source of energy in energetically demanding metabolic processes [20]. GLU levels, stored as glycogen, play a central role in metabolic demands of gametogenesis of bivalves [21], whereas TRIG, as the main form of lipids in marine bivalves, usually shows pronounced seasonal cycles [22]. Energy source CHOL is the principal sterol found in scallop tissues [23]. TAS, as a measure of overall antioxidant capacity, describes the dynamic equilibrium between different prooxidants and antioxidants in tissues, while SOD converts superoxide anion radical to hydrogen peroxide. The antioxidant GPx also catalyzes the decomposition of superoxide radicals into less toxic molecules [24]. The MDA level, proportional to the extent of lipid peroxidation, serves as a marker for oxidation of membrane lipids [13]. LDH regulates the cytosolic redox balance in glycolysis under anoxia and its activities are markers for anoxic conditions [25]. Carotenoids and astaxanthin are fundamental for maturation of bivalve gonads, have antioxidative properties, enhance tolerance to environmental stress, stimulate cellular growth and calcium transport over cell membranes [26]. Also, sequence analysis was used to assure proper classification of scallops sampled off western Istria coast as endemic Mediterranean scallops and to investigate possible haplotype diversity of the species from this part of eastern Mediterranean.

The aim of the study was thus to assess the impact of seasons and/or scallop tissues (gills, digestive gland, gonads, muscle) on biochemical homeostasis and the activity of defense system against oxidative damage in the unique environment of the Northern Adriatic Sea.

## Material and methods

All applicable international, national and/or institutional guidelines for the care and use of aquatic animals were followed and the procedures performed in the study were in accordance with the ethical standards of the institution. The ethical approval for bivalve molluscs is not required as per Directive 210/63/EU on the protection of animals used for scientific purposes.

### Study site

**Fig 1:**
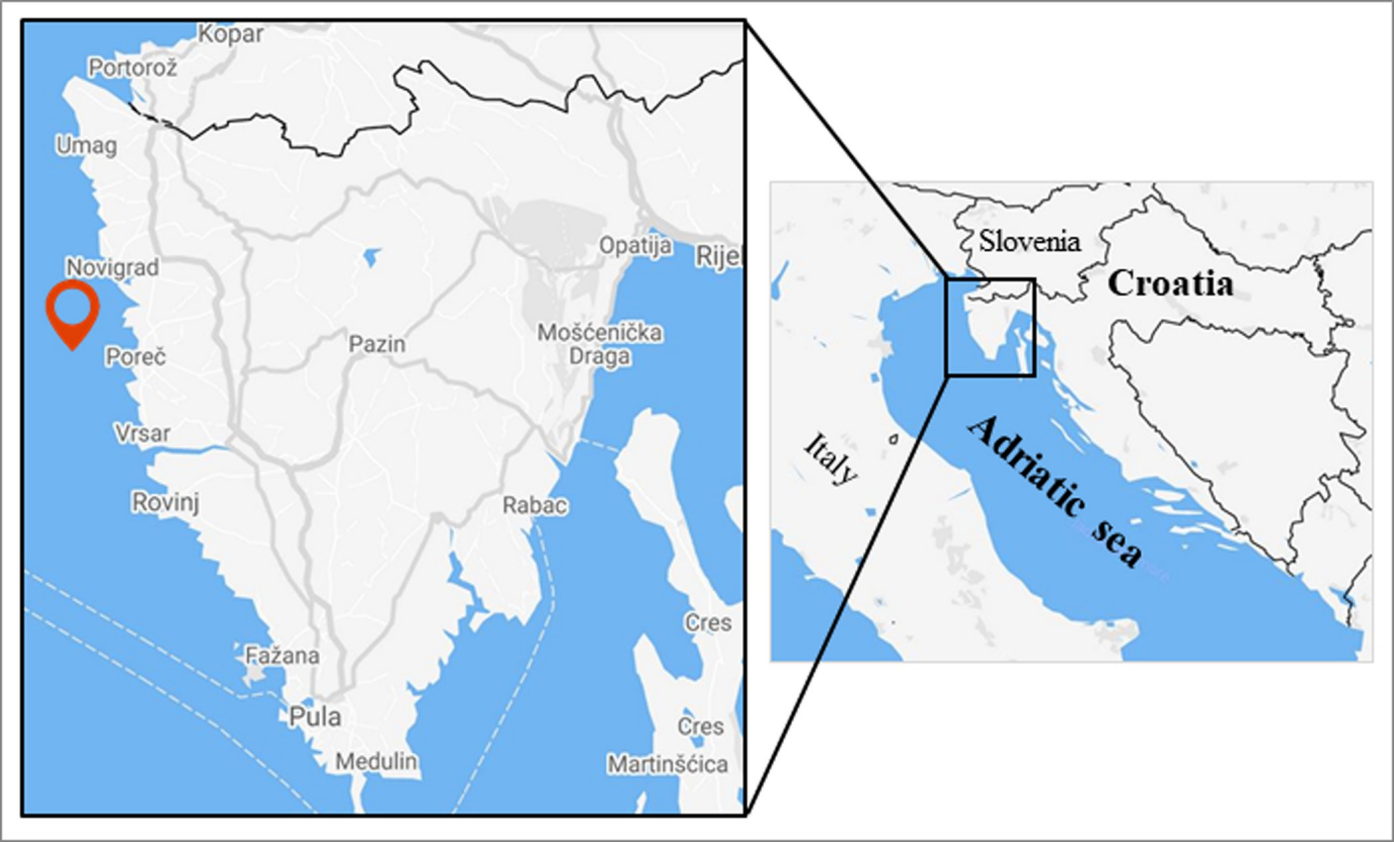
Map of the area. The label indicates the approximate location of the *P*. *jacobaeus* sampling, and the location of sea temperature, salinity and dissolved oxygen measurements.

The study was conducted on the Mediterranean scallop (*Pecten jacobaeus* L.) from the Adriatic Sea, collected 3 nautical miles off western Istria coast, Croatia (Fig 1). An undisturbed area abundant with *P*. *jacobaeus* was located using sonar and previous experience of fishermen. The area covered was at 45°13'15.3"N, 13°30'00"E with maximum depths of 40 m. The seabed was predominantly sandy. Sea temperature, salinity and dissolved oxygen (mean ± standard deviation) measured at depths of 15 m over seasons were as follows: in spring 16.87 ± 3.05°C, 36.07 ± 2.02 ‰, 5.41 ± 0.32 mg/L; in summer 23.4 ± 1.98°C, 38.3 ± 0.42 ‰, 4.77 ± 0.16 mg/L; in fall 20.9 ± 1.98°C, 38.1 ± 0.14 ‰, 4.99 ± 0.17 mg/L; in winter 12.55 ± 4.88°C, 38.15 ± 0.64 ‰, 5.89 ± 0.6 mg/L, respectively.

### Animals

*P*. *jacobaeus* were collected in spring, summer, fall, and winter. Scallops were retrieved as a side-catch by a bottom trawl with conventional diamond mesh netting used by fishermen to collect sole (*Solea vulgaris*) from the seabed. Upon hauling, the catch was emptied on deck and scallops were separated, sorted, enumerated, rapidly wet weighed, and inspected for external signs of mechanical damage. They were placed in sealed zip-lock PE bags and transported on shore on ice. Subsequently, they were frozen and kept at -86°C until further analyses. Before laboratory analyses, all samples were thawed and soft tissues were weighed individually upon blotting dry with paper.

Scallop tissues were analyzed over four seasons (n = 20 in spring, n = 20 in summer, n = 24 in fall, n = 20 in winter). Mean wet total weight was 38.00 ± 8.24 g, mean total length 115.96 ± 9.98 mm (spring); mean wet total weight 36.82 ± 5.92 g, mean total length 110.3 ± 4.58 mm (summer); mean wet total weight 21.12 ± 2.55 g, mean total length 93.12 ± 2.48 mm (fall); mean wet total weight 23.17 ± 2.8 g, mean total length 102.96 ± 5.11 mm (winter). The gonosomatic index (GSI) was calculated as [gonad weight/total tissue weight] × 100. The GSI was 4.65 ± 1.73 (spring), 3.99 ± 1.89 (summer), 7.37 ± 3.12 (fall), 5.51 ± 1.1 (winter).

### Chemicals

Thiobarbituric acid (CAS No. 504-17-6; TBA), trichloroacetic acid (CAS No. 76-03-9; TCA), butylhydroxytoluene (CAS No. 128-37-0; BHT) and malondialdehyde tetraethylacetat (CAS No. 122-31-6; MDA) were obtained from Sigma-Aldrich (St. Louis, MO, USA). Abbott commercial kits (Abbott, Germany) were used for biochemical tissue analyses and Randox commercial kits (Dublin, Ireland) were used for antioxidative tissue analyses. All other reagents used in this study were of chemical grade and commercially available.

### Analytical methods

Scallop tissues were separated after weighing (muscle, gonad, gills, digestive gland) and each tissue was weighed again and homogenized. Tissues were homogenized with Ultra-Turrax homogenizer (IKA, Germany) in cooled isotonic solution of 0.9% NaCl containing 0.1 mM phenylmethanesulfonyl fluoride (PMSF) as the protease inhibitor (1:3 w:v), on ice. Homogenates were centrifuged at 12 000*g* (20 min at 4°C) (Eppendorf 5804R, Germany). Supernatant was used for biochemical tissue analyses and lipid peroxidation, while pellet was used for electrochemical analyses. Tissue clippings for molecular analyses were performed on another set of scallops (10 specimens in spring).

### Biochemical tissue analyses and lipid peroxidation

Concentrations of total protein (TP), calcium (Ca), magnesium (Mg), glucose (GLU), triglyceride (TRIG), cholesterol (CHOL) and lactate dehydrogenase (LDH) activity in tissue homogenates were determined with Abbott commercial kits on biochemical analyzer Abbott Architect c4000 (Abbott, Germany). They were expressed as mg/g of tissue and U/g protein (LDH). The glutathione peroxidase (GPx) and superoxide dismutase (SOD) activities, as well as total antioxidative status (TAS) were measured with Randox commercial kits (Dublin, Ireland) on biochemical analyzer Abbott Architect c4000 (Abbott, Germany) according to manufacturer’s instructions. Enzyme activities were expressed as U/g of protein, and TAS as mmol/g of protein.

Lipid peroxidation determination was based on the formation of thiobarbituric acid reactive substances (TBARS), as a result of the reaction between malondialdehyde (MDA) and TBA under acidic condition. Lipid peroxidation was measured in all tissues (muscle, gonad, gills, digestive gland), in accordance with the method described by Babić et al. [27]. Briefly, after weighing, homogenizing and centrifuging, 250 μL of supernatant was added to 500 μL TCA-BHT reagent mixture (10% TCA, 0.01% BHT). After 15 sec of vortexing, samples were cooled for 15 min at 4°C and centrifuged for 10 min at 12 000*g*. The supernatant (750 μL) was mixed with 500 μL TBA and heated at 99°C for 30 min. The reaction stopped by cooling at 4°C, when the absorbency of supernatant was read at 535 nm using a FLUOstar OPTIMA plate reader (Infinite M200, Tecan, Austria). The amount of MDA-TBA complex was determined using an MDA standard curve and expressed as absorbance units per mg of tissue.

### Molecular analyses

Tissue samples from 10 individuals were stored in 96% ethanol until further processing in laboratory. Total DNA was isolated from mantle tissue using DNeasy Tissue Kit (Qiagen) following the manufacturer’s instructions. The amount and quality of obtained DNA was evaluated by electrophoresis on 1% agarose gel using ethidium bromide for visualization and documentation on DNA imaging system. DNA sequences of partial mitochondrial 18S rRNA were amplified using primers *Myt18S* F and *Myt18S* R [28]. All PCR amplifications consisted of 25-μL reaction volumes containing 0.2 mM of each dNTP, 0.2 μM of each primer, 1 U of Taq polymerase, 1.5 mM of MgCl and ~ 50 ng DNA. PCR cycling profile consisted of initial 10 min at 95°C, 35 cycles of 45 sec at 95°C, 45 sec at 50°C and 90 sec at 72°C, with final extension of 7 min at 72°C. PCR products were sequenced in both directions using the same primers as for PCR by Macrogen Europe (Amsterdam, The Netherlands). All sequences exhibited the same haplotype deposited in GenBank (accession number MT020507). The obtained haplotype was compared with published ones using BLAST search tool (http://blast.ncbi.nlm.nih.gov/).

### Electrochemical analyses: Extraction of carotenoids and voltammetry

Extraction procedure was adapted from literature [29]. Briefly, homogenized samples were weighted to approximate mass of 1.5 g per sample. On weighted samples 5 mL of dimethyl sulfoxide (DMSO, p.a.) was added and samples were sealed and placed in water bath at 50°C for 30 minutes. Every 10 minutes samples were vortexed for 15 sec. After elapsed time, samples were centrifuged for 5 minutes (4000 rpm). Supernatant was collected and remaining residue was re-extracted using different solvent, 5 mL of acetone. Samples were vortexed for 30 seconds, then centrifuged and supernatant was collected. This step was repeated until supernatant was colorless. Volumetric flask was then filled up to the point and placed in freezer until further analysis.

The applied voltammetric method was a relatively new approach named stripping voltammetry microprobe (SPV) which is based on a formation of a precipitate film of analyte on the electrode surface [30]. Namely, a precipitate of sample was formed onto the surface of paraffin impregnated graphite electrode (PIGE) by placing 5 μL of extract solution in acetone and allowing the solvent to evaporate in air. The precipitate was then analyzed by square-wave voltammetry (SWV) at optimal experimental conditions. Acetone was used as a solvent because of the low boiling point (56°C) so it could easily evaporate from electrode surface which is also advantage because extracted carotenoids are very sensitive to high temperature and oxygen. The working electrode was immersed in the electrolyte only during the voltammetric measurements. Less than 1 mm of the graphite rod was immersed into the electrolyte. SWV on modified PIGE was performed using a potential step increment of 2 mV, square wave amplitude of 50 mV and frequency of 100 Hz.

### Statistical analysis

All exploratory data analyses and statistical tests were performed using R v3.2.21 (R: A language and environment for statistical computing. URL: https://www.R-project.org/). Biochemical parameters were analysed as dependent variables of interest for each of four scallop tissues examined with gender of scallop and season of the year as explanatory variables. Parametric one-way ANOVA was used for data complying with assumptions of normality of residuals and homoscedasticity of variance. Data not complying with assumptions of ANOVA were analysed using the non-parametric Kruskal-Wallis test. Tukey's honest significant difference method was applied as a post hoc test following a significant ANOVA result while Dunn's post hoc test with Benjamini-Hochberg adjustment of p values was applied following a significant Kruskal-Wallis test result. Similarly, correlations of measured biochemical parameters were analysed using Pearson's correlation method for normally distributed data and Spearman's rank correlation for non-normaly distributed data. For correlation tests Bonferroni adjustment of p values was used. The level of significance was set at less than 0.05.

## Results

### Molecular analyses

Partial region of 18S rRNA was amplified in all studied samples with the size between 859 and 886 bp. All sequences exhibited the same haplotype (MT020507). Megablast search of NCBIs GenBank nucleotide database showed that our haplotype is identical with *Pecten jacobaeus* 18S rRNA partial sequence (AY070112) [31], *Pecten maximus* (L49053) [32], *Pecten maximus* isolate PmIV1 (EU660802) [33], *Pecten jacobaeus* isolate 17 (JQ611498) [34], *Pecten jacobaeus* isolate 18 (JQ611499) [34].

### Tissue biochemistry and lipid peroxidation

**Fig 2:**
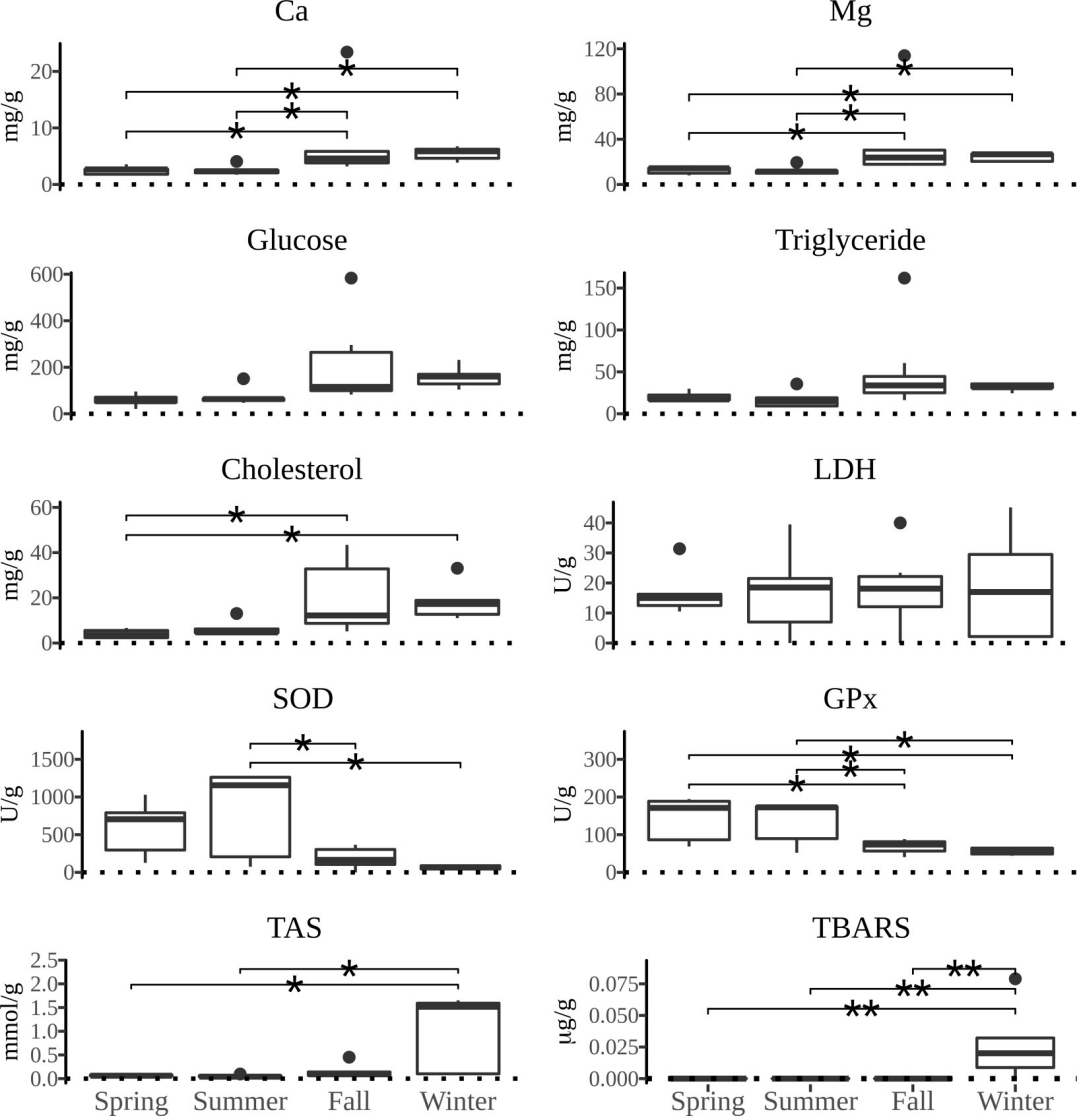
The boxplot distribution of biochemical parameters in muscle of *Pecten jacobeus* between seasons of the year. The bold bar shows the median value, the lower and upper hinges correspond to the first and third quartiles, and the whiskers extend from the hinges to the largest/lowest value no further than 1.5 times the inter-quartile range from the upper/lower hinge. Data beyond the end of the whiskers are outlying points and were plotted individually. The x-axes are dotted at zero to facilitate distinction from low values. Abbreviations: magnesium (Mg) calcium (Ca), lactate dehydrogenase (LDH), superoxide dismutase (SOD), glutathione peroxidase (GPx), total antioxidant status (TAS). The lines above boxplots show the significance level (* *p* < 0.05, ** *p* < 0.01, *** *p* < 0.001) of differences between seasons in the pairwise comparison. The endpoints of the lines mark two seasons with significant differences.

**Fig 3:**
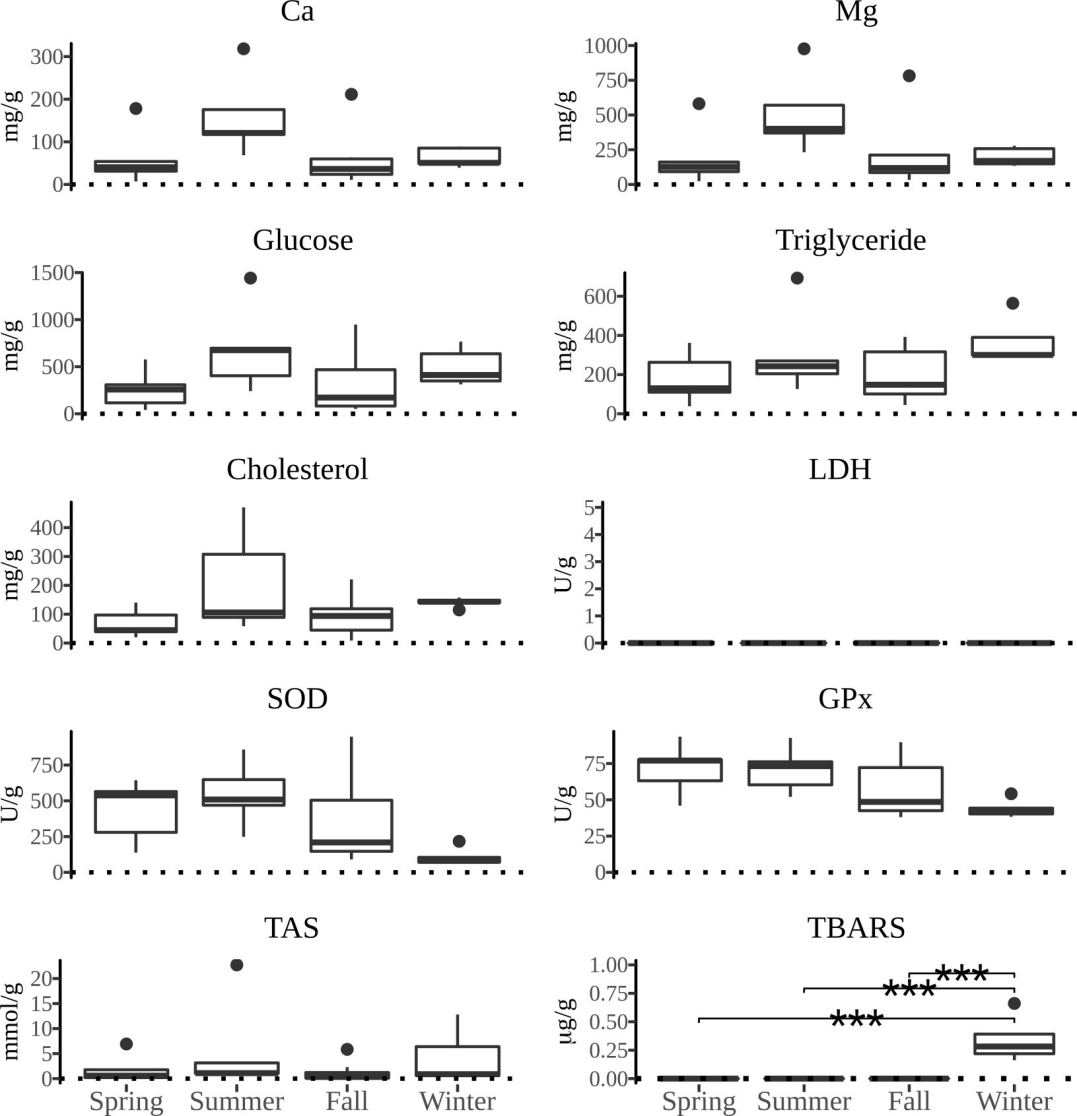
The boxplot distribution of biochemical parameters in gonads of *Pecten jacobeus* between seasons of the year. The lines above boxplots show the significance level (* *p* < 0.05, ** *p* < 0.01, *** *p* < 0.001) of differences between seasons in the pairwise comparison. The endpoints of the lines mark two seasons with significant differences.

**Fig 4:**
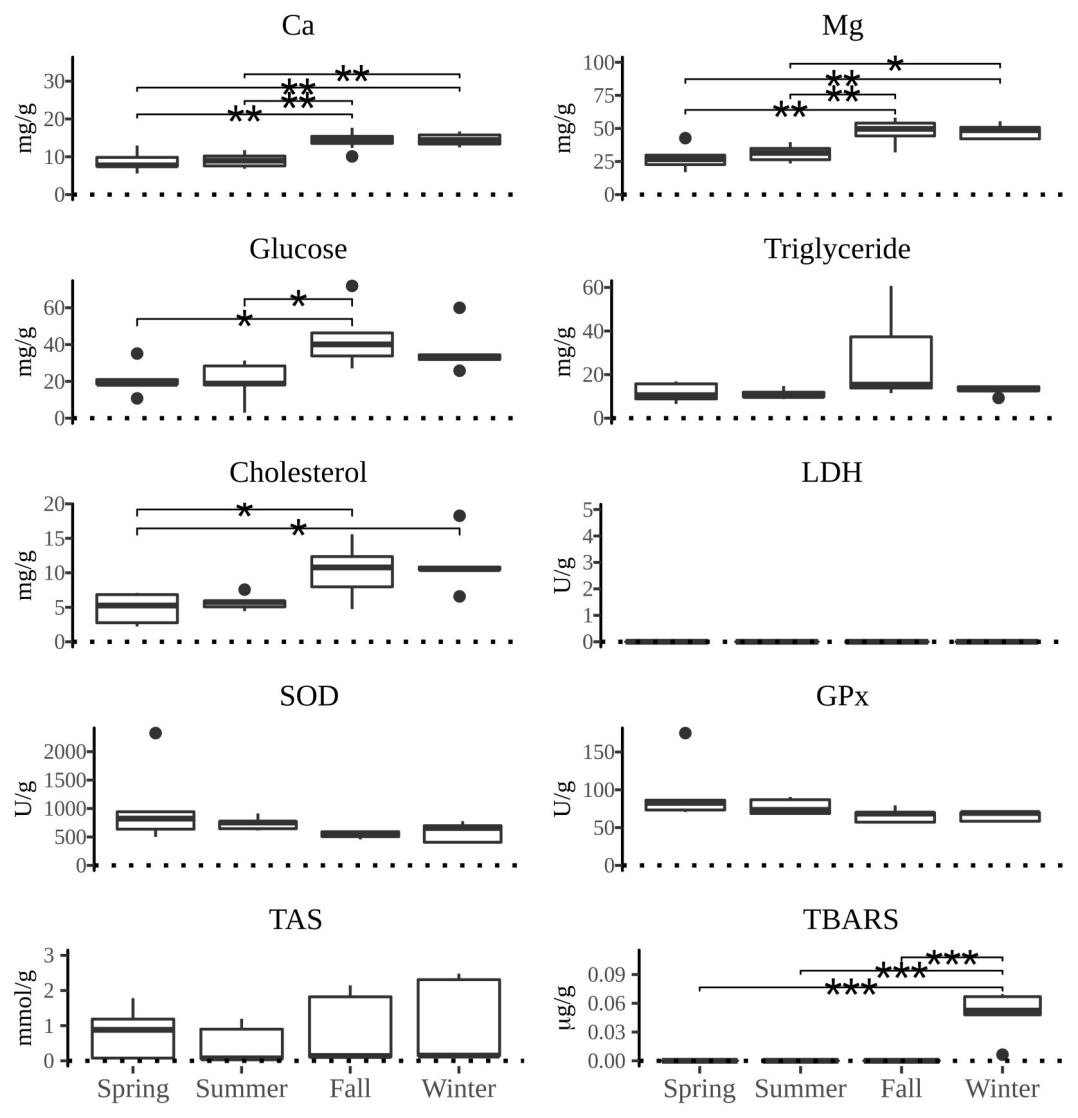
The boxplot distribution of biochemical parameters in gills of *Pecten jacobeus* between seasons of the year. The lines above boxplots show the significance level (* *p* < 0.05, ** *p* < 0.01, *** *p* < 0.001) of differences between seasons in the pairwise comparison. The endpoints of the lines mark two seasons with significant differences.

**Fig 5:**
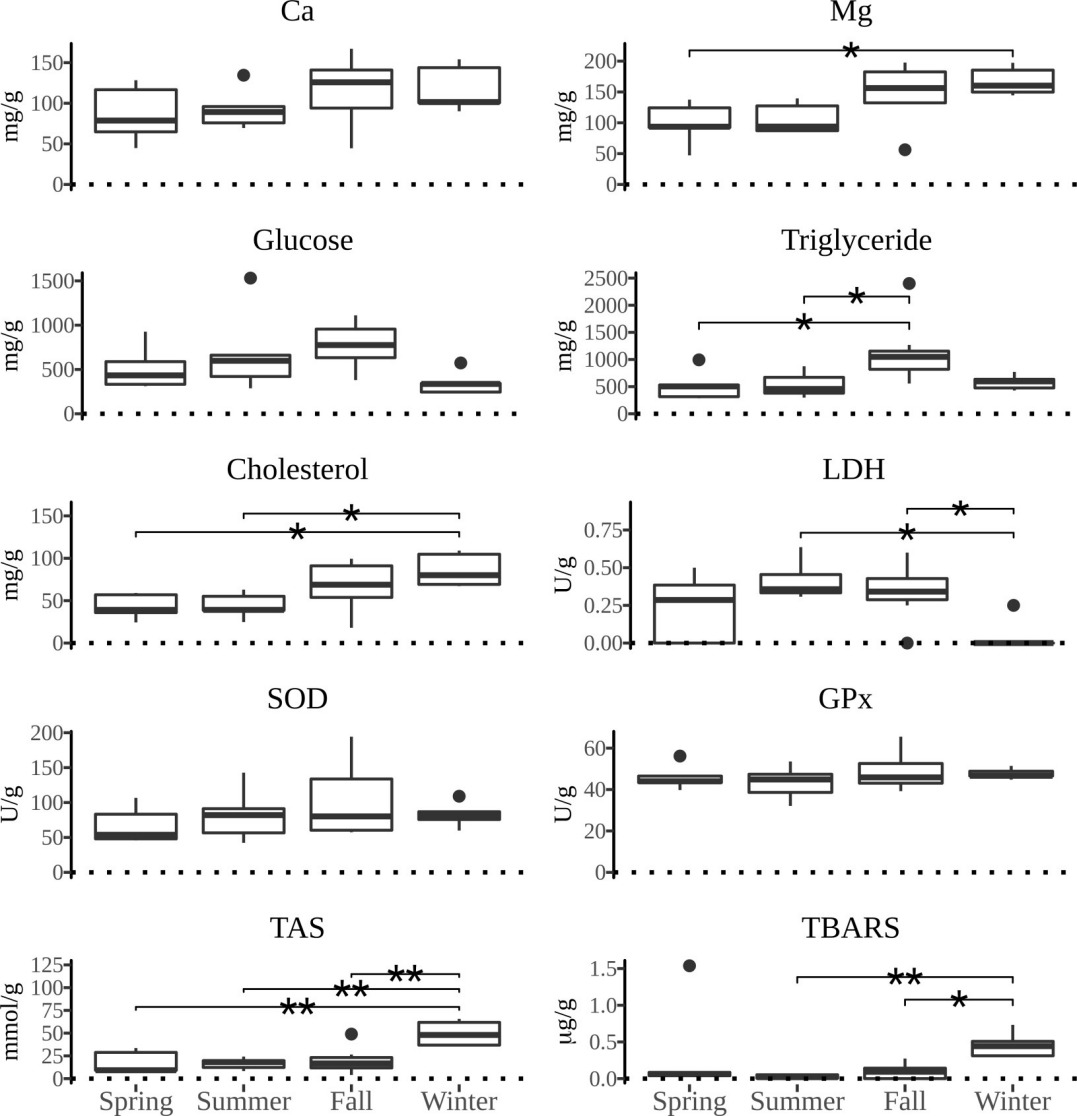
The boxplot distribution of biochemical parameters in digestive gland of *Pecten jacobeus* between seasons of the year. The lines above boxplots show the significance level (* *p* < 0.05, ** *p* < 0.01, *** *p* < 0.001) of differences between seasons in the pairwise comparison. The endpoints of the lines mark two seasons with significant differences.

All measured parameters of interest showed significant differences between the four types of tissue examined. The distribution of values of biochemical parameters measured and the activity of defense system against oxidative damage over the yearly seasons in four tissues of *P*. *jacobaeus* is depicted in Figs 2–5.

Calcium concentrations generally were the highest in summer and winter. The highest median concentrations for particular tissues were in summer in gonad and in winter in digestive gland. When comparing tissue Ca concentrations, the difference was the highest in gonad and digestive gland in relation to muscle and gills. Magnesium concentrations were the highest in summer and winter, although gonad had significantly higher concentrations than other tissues over the year, but particularly in summer (*p* < 0.05). Digestive gland Mg concentrations were comparable to gonads except for the summer season. Glucose concentrations were generally uniform over the seasons, except for the higher concentrations in summer, particularly in gonad, but were also elevated in fall in digestive gland. Triglyceride concentrations in digestive gland were significantly higher in fall compared to spring (*p* < 0.05) and summer seasons (*p* < 0.05), and higher than in all other tissues under examination. Cholesterol concentrations were significantly higher in gonad and digestive gland over other tissues (*p* < 0.05), and with a significant seasonal difference of winter having higher concentrations than summer (*p* < 0.05) and spring (*p* < 0.05)within digestive gland.

Total antioxidative status was highest in winter than all other seasons in all tissues except in gills where summer concentrations were higher than in winter, albeit not significantly. Generally, the winter concentrations were over 3-fold higher than in other seasons. The peak of TAS concentrations occurred in winter in digestive gland, and digestive gland showed significantly higher concentrations over all other tissues in all seasons (*p* < 0.05). The concentrations of SOD were the lowest in digestive gland compared to all other tissues examined and showed significant seasonal and gender differences. That is, in muscle tissue summer SOD concentrations were significantly higher than in fall and winter (*p* < 0.05) whereas in gills females had significantly higher concentrations than hermaphrodite scallops (*p* < 0.05). Similarly, GPx concentrations were significantly higher (*p* < 0.05) in females than hermaphrodites in all tissues except in digestive gland. Significant seasonal differences in GPx concentrations were observed in gills and muscle tissues (*p* < 0.05) with fall concentrations being higher than spring in gills whereas in muscles both spring and summer concentrations were higher than in fall and winter (*p* < 0.05). TBARS concentrations showed consistent tissue-wise seasonal differences with winter concentrations being significantly higher (*p* < 0.05) than in all other seasons for each tissue except for spring in digestive gland. However, the lack of significant difference between winter and spring TBARS concentrations in digestive gland was driven by a single outlier as removal of that datum returned a significantly different result of *p* < 0.05. Important to note is that except for the digestive gland TBARS concentrations in all tissues had zero values outside of winter months. The preponderance of zero values was also observed for lactate dehydrogenase in gills and gonads. A significantly higher (*p* < 0.05) concentrations of LDH were observed in both fall and summer in comparison to winter which was mostly due to 80% of winter results returning zero values.

Across all tissues examined, SOD and GPx had a positive correlation (*p* < 0.05) and both parameters were negatively correlated (*p* < 0.05) with TAS, Ca, Mg, glucose, cholesterol, and triglycerides in muscle tissue. Additionally, SOD was negatively correlated with glucose in gills. TAS was positively correlated with Mg and Ca in all tissues, with cholesterol in muscle and digestive gland, with glucose in muscle and digestive gland, and with TBARS in digestive gland. In addition to TAS, TBARS was positively correlated with Ca and cholesterol in digestive gland. Calcium was positively correlated with triglycerides in gonads and muscle, and Mg was positively correlated with triglycerides in all tissues except gills, and with glucose in gonads and muscle. Cholesterol, Ca and Mg were mutually positively correlated in all tissues. Cholesterol was also positively correlated with triglycerides in muscle and gonads. Glucose was positively correlated in all tissues with triglycerides, and with cholesterol and Ca in all except digestive gland.

### Voltammetry

**Fig 6:**
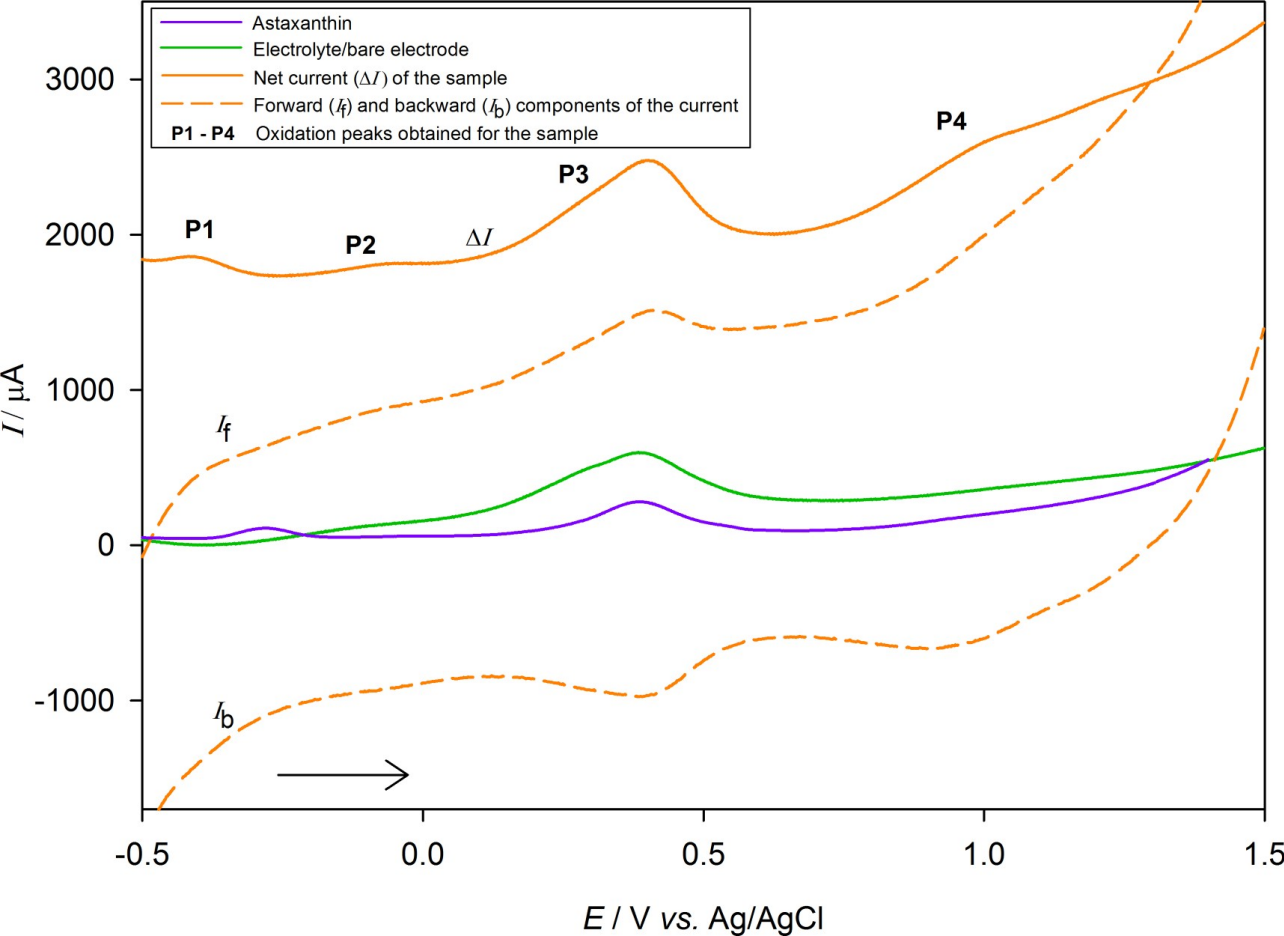
Square-wave voltammogram. The voltammogram corresponds to the oxidation of precipitate film of extract from digestive gland of Mediterranean scallops in winter, immobilized on PIGE and immersed in 0.1 M HClO_4_ solution. SWV of bare electrode (-∙-) in the same electrolyte is also shown. The frequency was 100 Hz, pulse amplitude was 50 mV and step potential was 2 mV. Peaks P1, P2 and P4 are typical for carotenoids.

Square-wave voltammetry was performed to determine possible carotenoids in different tissues of *P*. *jacobaeus*. Voltammetry analysis was performed on all tissues (gonad, muscle, gills and digestive gland), but no voltammetric peaks were observed when analyzing gonads, muscles and gills, *i*.*e*. the obtained voltammograms correspond to the one obtained for bare PIGE (Fig 6). The Fig 6 shows representative square-wave voltammogram of precipitate film of extract from digestive glands on surface of PIGE immersed into an aqueous 0.1 M HClO_4_ solution. This voltammogram is typical of the waveforms observed for all samples in this study. If the frequency is 100 Hz, the voltammogram consists of an irreversible peak P1 with net peak potential at -0.409 V *versus* Ag/AgCl/3M KCl. Irreversibility of this peak was confirmed by looking at backward component of current (*i*_b_). Further on, the peak P2 appearing at -0.070 V was followed by another reversible oxidation peak P3 at 0.389 V, and poorly developed quasi-reversible peak P4, with net peak potential at 0.969 V. The reversible peak P3 was the result of using perchloric acid as an electrolyte in combination with PIGE, which was confirmed by measuring current of bare PIGE in this aqueous electrolyte (Fig 6). The representative peaks varied over the seasons, with their maxima in winter months.

## Discussion

The study describes the impact of seasons on biochemical parameters relevant for ionic homeostasis, reproduction control, protein catabolism, and energy source, as well as on the activity of oxidative damage defense system in four scallop tissues. The North Adriatic seawater has seasonal variations of salinity, temperature and water quality [35], which might influence the physiological and biochemical responses of bivalves. Strong seasonal variability triggers atmospheric forcing that subjects the sea to intense fluxes of heat, water and buoyancy, while the Po river input supplies the basin with buoyancy fluxes, playing a major role in the stratification of the water column throughout the seasons. The stratification of the water column is more intense in summer due to the inflow of light river Po surface water, and salty warm water at the bottom [36]. The observed decrease in GSI in summer might thus be related to the spawning or resorption of gametes due to increased temperatures and phytoplankton abundance. It was noted that in the Northern Adriatic *P*. *jacobaeus* gonads are mature from May to July and November and February [37], which correlates well with the GSI peak in fall in this work, as well as the GSI increase in conjunction with decreasing water temperature in the work of Takahashi and Mori [38]. The *P*. *jacobaeus* length of 100 mm and over, as found in most of our samples, indicates to specimens of over three years of age [8].

The Mg and Ca relationships in shells enable the analysis of salinity and temperature variations during the shell formation [39]. In scallop tissues under study their mutual relationship had significant correlations. Due to the importance of Ca and Mg for structure and function of the striated muscle in scallops, their relatively low levels in muscle tissue in this work was not expected. However, Ca is known to be moved by hemocytes from the digestive gland to other tissues [40], and was also found in high levels in gonads. Magnesium, on the other hand, plays a regulatory role in oxidative processes, and is considered an essential cofactor of GSH synthesis. Indeed, in this work it was found that Mg and TAS have significant correlation in all tissues examined. Magnesium decrease leads to reduction in glutathione levels and intensifies the production of ROS [41]. Indeed, we found it on the lowest in muscle tissues in spring and summer, just as the GPx activities.

Scallops exhibit cycles of energy storing and usage, closely related to gametogenetic cycles, therefore biochemical composition changes of body components reveal which substrate participates in energy metabolism [42]. In periods of lower energy demands, the reserves of energy usually directed to gametogenesis, remain in the muscle. Although an inverse relationship between scallop adductor muscle energy contents and GSI was previously established in Mexico [43], *P*. *jacobaeus* from Northern Adriatic in our work demonstrated muscle energy maxima in correlation with the maximum gonosomatic index in fall, when diatoms and coccolithophorids thrive. Thus, cholesterol and glucose in adductor muscle significantly varied (*p* < 0.01) in our spring *vs*. winter specimens. Nutrient reserves stored in adductor muscle and digestive gland are utilized to assist in gamete development, and may vary with availability of food supply in the particular environment. If the availability of food is sufficient for gametogenic and somatic growth, catabolism of muscle reserves does not take place [42]. Also, we showed that levels of GLU, TRIG and CHOL in four tissues under examination were positively correlated and were predominant in digestive gland and gonad. These reserves are used for basic metabolism in months when food is scarce, but also for gametogenesis [42]. Digestive gland is the main site for lipid reserves both in scallops and mussels, where the main type of lipids consists of neutral lipids [44]. The Adriatic *P*. *jacobaeus* levels of GLU and TRIG were the highest in fall, CHOL in summer (gonads) and winter (digestive gland). In that regard, fall and winter are the peak seasons for diatoms and coccolithophorids in Istria, while late spring is the peak season for development of other phytoplankton species serving as food for the Mediterranean scallop [35]. Since the region is under the influence of the river Po water inflow, thus the phytoplankton abundance depends on the precipitation and snow melting in the Alps in fall and spring, respectively.

The peroxyl radicals in tissues are counteracted by the cellular antioxidant defenses, thus the damage incurred depends on the ratio of formation of oxidant species and antioxidant defense mechanisms, consisting of low molecular weight scavengers and antioxidant enzymes [45]. The seasonal variation of antioxidant enzyme activities in this work was inversely related for TAS and TBARS *vs*. GPx and SOD. The winter maxima of TAS and TBARS *vs*. summer maxima of GPx and SOD in digestive glands indicate to a decrease in antioxidant defense during cold months, and are related to the accumulation of lipid peroxidation products (such as malondialdehyde) in digestive gland of scallops, also observed by Viarengo et al. [46]. Winter temperatures and high levels of oxidative damage contributed to the significant (*p* < 0.001) muscle mass reduction of *P*. *jacobaeus*. Although decreased temperatures often imply higher levels of dissolved oxygen, affecting peroxyl radical production, lipid peroxidation in scallops [47] was enhanced also by increased temperatures. The SOD activities elevated in warm temperatures in digestive gland and gills of mussels [15], although in our work in summer they increased mostly in gonad, while in gills they peaked in winter. However, the increases in antioxidant enzyme activities could be associated with, and are partially responsible for the decreased TBARS levels [15]. In particular, the decrease of TBARS concentration in summer, linked with the low GSI, could have occurred after a spawning event of *P*. *jacobaeus* in the Northern Adriatic.

The decrease of antioxidant defense levels could also be linked to the varying metabolic status of scallops, depending not only on gonad maturation, but also on accessibility of food. A feeding behavior of scallops may have thus contributed to high levels of GPx in digestive gland and gonad in summer, since *P*. *jacobaeus* rely upon suspended detrital material and phytoplankton as their food source. Adult scallops are known for ingesting relatively large particles due to the absence of gill sorting mechanisms for particles [48]. Therefore, high GPx levels may have been influenced by *P*. *jacobaeus* diet components [45]. Its difference in various tissues was previously also demonstrated [49], up to 24-fold.

Voltammetry successfully recognized carotenoids extracted from digestive gland of scallops, in agreement with Čižmek et al. [50], conducting analyses of three different carotenoids. Although signals obtained were in accordance with voltammeric responses for carotenoid astaxanthin, more than one carotenoid was present in the samples. Voltammetry thus enabled rapid screening of carotenoids in *P*. *jacobaeus* and gave insight into their accumulation in different tissues. As carotenoids were accumulated in digestive gland, it can be concluded that most of them were ingested. Their seasonal variations might be related to different food availability and fluctuations of their biomass typical of Istrian Adriatic [46]. Ingestion of pigments also showed a rapid response to seasonal changes of digestive gland pigment content of *P*. *maximus*, which responded with a small time-lag to increasing phytoplankton concentration at the sediment-water interface [51]. Diatoms in the Northern Adriatic are the most abundant plankton regarding the pigment contents [52], particularly carotenoids, which explains their high SWV peaks in fall and winter. Also, their seasonal involvement in the scavenging of ROS, singlet molecular oxygen, and peroxyl radicals, contributes against lipid peroxidation, bearing in mind that excess light might lead to photoinhibition and (again) formation of the ROS [53]. It was shown that carotenoid content in bivalves has a significant positive correlation with their antioxidant defense system under environmental stress, as the high carotenoid content enables high resistance to marine pollution and abiotic stressors [54]. In this work, SW voltammetry was for the first time used for qualitative analysis of carotenoids from *P*. *jacobaeus* tissues and proved as a straightforward, sensitive and chemical species-selective technique for their measurement.

Furthermore, the molecular analysis of the samples revealed absence of haplotype diversity among Mediterranean scallops sampled on the Istrian part of the Adriatic Sea (eastern Mediterranean). This finding is in concordance with previous studies which detected no intraspecific variability and low interspecific variability of the 18S rRNA region pointing out this region as a possible molecular marker for the genetic identification of bivalve species [28,33,55]. However, 100% identity of *P*. *jacobaeus* and *P*. *maximus* indicate a low-resolution power of 18S as a molecular marker for discrimination of *P*. *jacobaeus* from *P*. *maximus*.

The present work on identical Mediterranean scallop haplotypes demonstrates the first study of their biochemical properties, antioxidant defenses, and free radical scavengers during the yearly seasons in the Northern Adriatic, off Istria. Variations of these responses provide tools for assessments of impact of environmental outliers on scallop physiological status. Although the increased susceptibility to oxidative stress could be attributed to winter temperature impact towards accumulation of lipid peroxidation products, other factors, such as the availability of food supply, might counteract that effect, particularly in the view of various tissues under examination. Also, water salinity might prove as a contributing factor in variations of biochemical parameters over the seasons, particularly taking in consideration spring *vs*. summer endpoints. It is thus important to establish ranges of seasonal variability for measured parameters for different scallop tissues in order to obtain comparable information on its effect on this highly exploited species endemic to the Mediterranean. That is particularly important as the seawater alters its salinity, temperature and water quality not only in relation to seasons, but also by the river Po influx, which might influence the physiological and biochemical responses of scallops in the Northern Adriatic Sea.

## Supporting information

S1 Table Raw data: Sea water temperature, salinity, oxygen. (XLSX)

S1 Fig Correlogram of muscle tissue. All correlations between all measured parameters in muscle tissue of *Pecten jacobaeus* are shown with colours indicating the strength of correlation.
(PNG)

S2 Fig Correlogram of gonad tissue. All correlations between all measured parameters in gonad tissue of *Pecten jacobaeus* are shown with colours indicating the strength of correlation.
(PNG)

S3 Fig Correlogram of gill tissue. All correlations between all measured parameters in gill tissue of *Pecten jacobaeus* are shown with colours indicating the strength of correlation.
(PNG)

S4 Fig Correlogram of digestive gland tissue. All correlations between all measured parameters in digestive gland tissue of *Pecten jacobaeus* are shown with colours indicating the strength of correlation.
(PNG)
