# Supplementary figures and images for: Seasonal antioxidant and biochemical properties of the Northern Adriatic Pecten jacobaeus
Source: PLoS One. 2020 Mar 18;15(3):e0230539. doi: 10.1371/journal.pone.0230539 (PMC7080251; doi:10.1371/journal.pone.0230539)

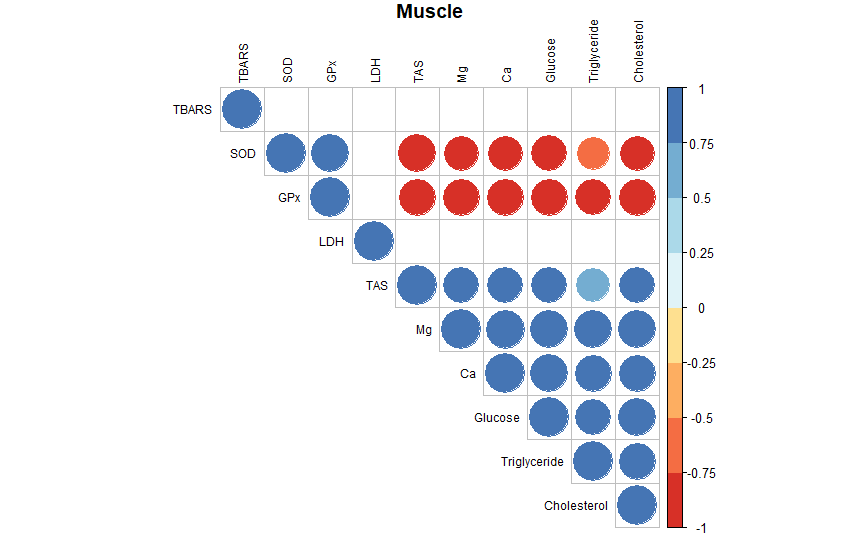

Supplement: S1 Fig — All correlations between all measured parameters in muscle tissue of Pecten jacobaeus are shown with colours indicating the strength of correlation. (PNG) [file pone.0230539.s002.png]

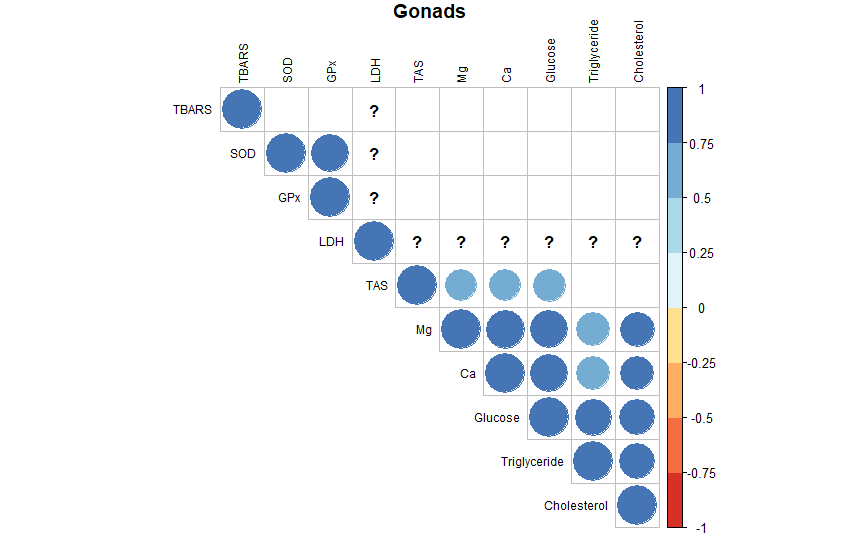

Supplement: S2 Fig — All correlations between all measured parameters in gonad tissue of Pecten jacobaeus are shown with colours indicating the strength of correlation. (PNG) [file pone.0230539.s003.png]

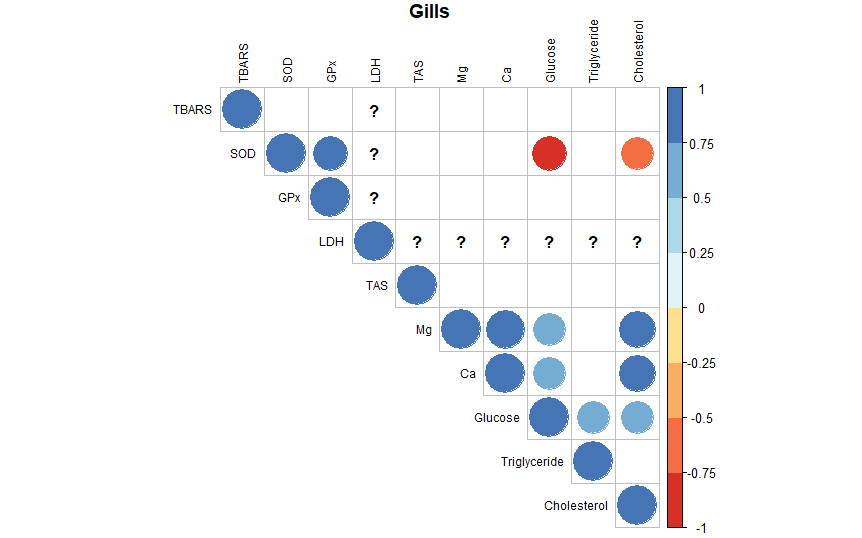

Supplement: S3 Fig — All correlations between all measured parameters in gill tissue of Pecten jacobaeus are shown with colours indicating the strength of correlation. (PNG) [file pone.0230539.s004.png]

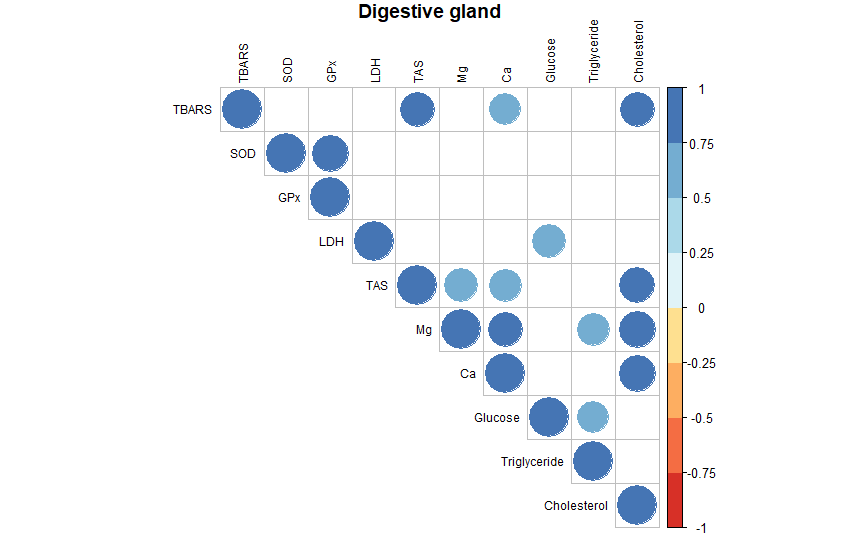

Supplement: S4 Fig — All correlations between all measured parameters in digestive gland tissue of Pecten jacobaeus are shown with colours indicating the strength of correlation. (PNG) [file pone.0230539.s005.png]
